# Infection with *Borrelia burgdorferi* Increases the Replication and Dissemination of Coinfecting Powassan Virus in *Ixodes scapularis* Ticks

**DOI:** 10.3390/v14071584

**Published:** 2022-07-21

**Authors:** Charles E. Hart, Frank A. Middleton, Saravanan Thangamani

**Affiliations:** 1 Department of Microbiology and Immunology, Upstate Medical University, Syracuse, NY 13210, USA; hartch@upstate.edu; 2 SUNY Center for Vector-Borne Diseases, Upstate Medical University, Syracuse, NY 13210, USA; 3 Institute for Global Health and Translational Sciences, Upstate Medical University, Syracuse, NY 13210, USA; 4 Department of Biochemistry and Molecular Biology, Upstate Medical University, Syracuse, NY 13210, USA; middletf@upstate.edu

**Keywords:** *Borrelia burgdorferi*, Lyme disease, Powassan virus, ticks, *Ixodes scapularis*, coinfection (min. 5–max. 8)

## Abstract

Powassan virus (POWV) is a tick-borne neuroinvasive flavivirus endemic to North America. It is generally transmitted by the tick, *Ixodes scapularis*. This species also transmits *Borrelia burgdorferi*, the causative agent of Lyme disease. Infection with *B. burgdorferi* can result in arthritis, carditis, and neuroborreliosis. These pathogens experience sylvatic overlap. To determine the risk of human exposure to coinfected ticks, the interactions between POWV and *B. burgdorferi* are assessed in laboratory-infected *I. scapularis*. Adult male and female *I. scapularis* ticks are orally inoculated with either both pathogens, POWV only, *B. burgdorferi* only, or uninfected media. After twenty-one days, the ticks are dissected, and RNA is extracted from their midguts and salivary glands. In infected midguts, the quantity of POWV in coinfected ticks was elevated compared to those with only POWV. In addition, the salivary glands of ticks with infected midguts had increased POWV dissemination to those with only POWV. RNA sequencing is performed to identify the potential mechanism for this pattern, which varies between the organs. *Ixodes scapularis* ticks are found to be capable of harboring both POWV and *B. burgdorferi* with a benefit to POWV replication and dissemination.

## 1. Introduction

Powassan virus (POWV) is a tick-borne, neurotropic flavivirus endemic to North America and Russia. It exists in two sylvatically distinct but serologically identical lineages, with Lineage I reservoired in groundhogs (*Marmota monax*) and striped skunks (*Mephitis mephitis*), vectored primarily by the groundhog tick (*Ixodes cookei*) or squirrel tick (*Ixodes marxi*). The Lineage II POWV is reservoired in *Peromyscus* spp. mice and vectored by the deer tick (*Ixodes scapularis*) [1,2,3], although shrews (Soricidae) have been identified as another possible reservoir [4]. The virus replicates in the salivary glands of an infected tick and can be transmitted in as little as 15 min upon attachment to a mammalian host [5].

Symptomatic POWV infection begins with a febrile illness that, in some cases, may be self-resolving. However, in an unknown proportion of cases, it progresses to a neuroinvasive state that involves meningitis, encephalitis, and, occasionally, meningoencephalitis [6,7,8,9]. This leads to coma and a mortality rate of up to 17% [10], with survivors experiencing long recovery times and 55% experiencing permanent neurological sequelae [11] consisting of cognitive and motor defects.

POWV shares a geographical range, reservoir, and vector with *Borrelia burgdorferi*, the causative agent of Lyme disease. Lyme disease is a tick-transmitted illness that can cause erythema migrans and febrile illness followed by oligoarthritis [12], and carditis [13], involving disruption of the heart’s electrical system [14]. Additionally, neuroinvasion of spirochetes can also result in radiculitis [15], palsy, vertigo, Barnsworth’s syndrome, and invasion of the brain resulting in cognitive and depressive disorders [16] as well as changes in the mental state [17]. Treatment is possible with antibiotics, although symptoms may persist due to an autoimmune response from cross-reactive antibodies against the spirochetes, called post-treatment Lyme disease syndrome [18].

Unlike POWV, which has caused between 20 and 40 cases per year since 2016 [10], Lyme disease is common, causing 16,000–30,000 reported cases per year in the United States, primarily in the Northeast and the upper Midwest [19]. Field studies in endemic areas have suggested that POWV is rare in wild ticks, existing at a rate of 5% or less [20,21,22]. *B. burgdorferi*, however, is common, with a 20–60% infection rate [23,24,25,26]. If the pathogens exist in the same geographical area and assort independently within the tick without interaction between the two, then any POWV-infected tick would have an equal chance of being infected with *B. burgdorferi* as all ticks in the same area. Therefore, while the actual rates of POWV in ticks are low, the majority of this minority population is likely to be co-infected with *B. burgdorferi*.

This potential ability for a single tick to transmit both POWV and *B. burgdorferi* is dependent on internal pathogen interactions within the tick. Such interactions have been observed for other pathogens as well. *B. burgdorferi* has also been shown to enhance the acquisition of *Babesia microti*, raising the ecological threshold for *B. microti* emergence [27]. Less information is known about interactions with viruses. However, an in vitro study found that the presence of *B. burgdorferi* in culture can enhance the replication of the Semliki Forest virus, an alphavirus [28].

Natural cases of co-infection have been observed, but always at a low rate. In New York and Connecticut, one study found *B. burgdorferi*-POWV co-infection rates as high as 2%, with a rate of up to 67% *B. burgdorferi* infection and a 1–2% rate of POWV infection [25]. Moreover, in New York, a study identified a 2% rate of co-infection with a base POWV rate of 3% and a *B. burgdorferi* rate of 55% [26]. In a study in Wisconsin, the rate of co-infection was 1%, with an overall POWV rate of 5% and a *B. burgdorferi* rate of 22% [29]. Human cases of apparent co-exposure have been identified as well, with a study of seven patients with anti-POWV IgM tested and six showing anti-*B. burgdorferi* IgM, suggesting simultaneous infection [30].

Whether these represent instances of long-term, consistent sylvatic maintenance or a transient intersection is unclear, and adequate information does not exist to assess the human risk of encountering a tick capable of transmitting both POWV and *B. burgdorferi*. While human co-infection may result from exposure to multiple infected ticks rather than a single co-infected tick, the high probability of POWV-infected ticks also containing *B. burgdorferi* suggests that single-tick inoculation may be a common mode of co-exposure. Understanding the interaction between the pathogens within the tick, therefore, is necessary for a complete understanding of the role of co-transmission and the development of human disease.

Within the tick, both *B. burgdorferi* and POWV first encounter the midgut when consumed during feeding. *Borrelia burgdorferi* then remains relatively restricted to the lumen of the midgut until feeding [5], while POWV disseminates throughout the tick, eventually infecting all tissues prior to feeding. The pathogens do not interact directly and exist in different niches within the tick. Therefore, any reactions are a result of intersecting anti-spirochete and anti-POWV immune responses. Ticks, similar to all arthropods, have an advanced innate immune system [31], but lack an adaptive immune response. The response to bacteria is dependent on neutrophil-like and macrophage-like cells, specifically granulocytes or hemocytes, respectively [32], which utilize phagocytosis and the generation of reactive oxygen species to combat cellular pathogens. These cells freely migrate through the tick’s open circulatory system and are activated by pathogen-sensing molecules such as Toll [33] and CD36/HISRB [34]. These may also respond to viral infection [33], although much of the tick’s antiviral response involves using the RNAi inhibition system to degrade cytoplasmic viral RNA [31,33].

Under normal circumstances, ticks acquire pathogens from vertebrate sources during feeding. These pathogens persist as the tick molts to its next life stage and are transmitted during subsequent feedings. Feeding induces substantial physiological and transcriptional changes within the tick, an aspect that is ignored by the artificial capillary-inoculation method used in this experiment. The capillary inoculation method places pathogens through the alimentary canal of the tick, resulting in infection of the midgut to mimic the natural course of infection. Here, the goal is to model unfed, questing adult ticks that have fully completed blood digestion and molting from a nymphal feeding. This is intended to examine pathogen interactions only at this specific portion of the pathogen’s transmission, with acquisition and transmission separated for future studies.

Infection of ticks with *B. burgdorferi* or tick-borne flaviviruses has previously been shown to induce a transcriptional response in both the tick midguts and salivary glands. Infection of ticks with *B. burgdorferi* or its European analog, *B. afzelii,* induces alterations to tick metabolism as well as to the tick’s immune and antimicrobial response [35,36,37]. Changes have also been observed in the salivary glands in response to spirochete infection [38] as well as tick-borne flaviviruses [39,40]. Additional studies on tick cell lines have indicated similar immune responses to flaviviral infection [41].

It is difficult to deduce whether this occurs from field studies alone due to the low observed rate of POWV and the lack of quantification data that could be used to assess altered replication rates. Further, analysis of the transcriptome can assist in identifying the exact intersection point of the anti-pathogen interactions to determine the source of any inhibitory or synergistic effects within the tick immune response.

This information will assist in predicting the possibility of human co-exposure to both the Powassan virus and *B. burgdorferi* in regions endemic to both pathogens.

## 2. Methods

### 2.1. Tick Origin and Maintenance

The ticks used for this experiment were colonized at the University of Texas Medical Branch, as previously described [5,42]. The ticks were maintained at 22 °C with greater than 95% humidity. This was accomplished by using sealed glass desiccators with deionized water placed in the bottom reservoir and glass vials containing the ticks kept on the central plate over it. A 12 h light/12 h dark cycle was maintained by using LED lights in large incubators where the glass desiccators were held [42].

### 2.2. Virus Culture

Powassan virus (LB strain, M794/TVP-16654) was acquired from the University of Texas Medical Branch (UTMB) World Reference Center for Emerging Viruses and Arboviruses (WRCEVA, UTMB) in lyophilized form. The virus was reconstituted with sterile deionized water and cultured on Vero cells. The media used for the growth of these cells consisted of Minimum Essential Media (MEM, Gibco, Grand Island, NY, USA) with 8% heat-inactivated fetal bovine serum (FBS, Thermo Scientific, South Logan, UT, USA), 1% non-essential amino acid solution (NEAA, Gibco, Manassas, VA, USA), and 1% penicillin-streptomycin antibiotic solution (Gibco, Manassas, VA, USA). For viral culture, the concentration of FBS was reduced to 2%. This medium was used for the first five passages of the virus. The sixth passage was prepared without antibiotics to prevent potential inhibition of the growth of *B. burgdorferi* during infection media preparation. For this final passage, the virus was cultured on Vero E6 cells grown in culture media identical to the type used for previous passages except for having been prepared without added antibiotics. This was grown for four days, aliquoted, and stored at −80 °C until use.

The concentration of the virus was quantified using a focus-forming assay. In brief, this involved plating a serial dilution of the virus on confluent Vero E6 cells in six-well plates. These were allowed to settle for one hour before being overlaid with media that consisted of Opti-MEM base media (Gibco, Grand Island, NY, USA) with 3% FBS, 1% glutamate solution (Gibco, Manassas, VA, USA), and 0.8% methylcellulose (Fisher Bioreagents, Fair Lawn, NJ, USA), fully dissolved. The plates were then returned to the incubator for four days. The overlay was then removed, and the plates were fixed with 1:1 methanol/acetone (Fisher Bioreagents, Fair Lawn, NJ, USA). After drying, these were stained using anti-POWV antibodies (WRCEVA, UTMB) with HRP-conjugated goat-anti-mouse (Invitrogen, Waltham, MA, USA) as the secondary antibody. The plates were developed using an ImmPACT AEC kit (Vector Labs, Burlingame, CA, USA), and the foci were counted to determine the viral quantity in the original stock.

### 2.3. Bacterial Culture

A frozen stock of *B. burgdorferi* (strain B31) was obtained from the Center for Disease Control and Prevention (CDC). The culture medium used consisted of BSK-H media (Sigma) supplemented with 6% Fraction-V BSA (Fisher Bioreagents, Fair Lawn, NJ, USA). The solution was sterilized using a 0.22 μm syringe filter (MERK Millipore, Cork, Ireland). Supplemented culture media were prepared immediately prior to use for each passage of bacteria. Material from the initial stock was scraped and placed in a tube containing freshly prepared media, then incubated at 34 °C with 4% CO_2,_ and the lid of the tube loosened to allow gas exchange. No agitation was used, and the culture tubes were kept stationary during incubation for 5–8 days. At the conclusion of the growth period, the bacteria were mixed and counted using a hemocytometer (Thermo Fisher Scientific, Grand Island, NY, USA) to determine the bacterial concentration of the culture.

### 2.4. Tick Inoculation

*B. burgdorferi* spirochetes were centrifuged at 3000 rcf for 15 min at 8 °C. The supernatant was removed, and the pellet resuspended in a mixture of equal parts defibrinated sheep’s blood (Colorado Serum Company, Denver, CO, USA) and freshly prepared culture media (BSK-H with 6%FBS) to a concentration of 3 × 10^8^ cells/mL. Some of this blood/media mixture was also reserved for the preparation of the uninfected negative control.

MEM media was prepared to the same specifications as viral culture media (2% FBS, 1% NEAA). The virus was thawed, and the stock diluted to a concentration of 2.32 × 10^5^ FFU/mL. Uninfected control media was created as a mixture of equal parts uninfected MEM media and 1:1 BSK-H media and blood. *Borrelia*-only inoculant consisted of *B. burgdorferi* in BSK-H/blood media at 3 × 10^8^ cells/mL mixed with uninfected MEM media, while POWV-only inoculant consisted of BSK-H/blood without *B. burgdorferi* combined with viral stock in MEM media at a viral concentration of 2.32 × 10^5^ FFU/mL. Co-infection media was generated as a mixture of both pathogen solutions.

Ticks were infected by first attaching them in an inverted position to double-sided tape. Glass capillaries (10λ) were filled with infection fluid and affixed over the tick mouthparts such that they surrounded both the hypostome and palps. The ticks were then covered with damp paper towels to maintain humidity and allowed to incubate at room temperature for one hour, checked periodically to confirm that they had not extricated themselves from the tape or dislodged the capillaries.

At the completion of the feeding, the capillaries were removed, and the ticks gently removed from the tape. They were then washed in 70% ethanol and dried before being placed in fresh glass vials and returned to their desiccator. The ticks do not engorge during this process and do not appreciably change in size or appearance after inoculation.

The ticks were maintained at 24 °C and at 95% humidity, with a daily light/dark cycle of twelve hours light to twelve hours darkness. The ticks remained in this location with minimal disturbance for 21 days.

### 2.5. Tick Dissection and RNA Extraction

Ticks were dissected at 21 days post-infection (DPI), and midgut and salivary glands were placed in respective containers with 500 μL Trizol (Ambion, Carlsbad, CA, USA). The remainder of the body was also placed in a tube with 500 μL Trizol. Tools were cleaned in 70% ethanol between uses. Individual test groups were dissected separately, starting with the control group.

The Trizol mixtures were stored at 4 °C for 24 h for pathogen neutralization and then stored at −20 °C until used for RNA extraction. For RNA extraction, the samples were first thawed. For homogenization, a 4.5 mm zinc-plated steel bead (Daisy Outdoor Products, Rogers, AR, USA) was then placed in each tube and the contents processed with a Qiagen Tissue Lyser II machine for 5 min at 30 Hz.

After homogenization, the steel beads were removed using a magnet. Then, 200 μL chloroform was then added to each tube, and the tubes vortexed to emulsify. The tubes containing Trizol/chloroform emulsion were then centrifuged at 12,000 rcf for 15 min at 4 °C to separate the aqueous and organic layers. The aqueous layer from each tube was removed and placed in a new tube, then diluted 1:1 with 70% ethanol. The resulting solution was mixed and used as the starting material for RNA extraction using a Qiagen RNAeasy extraction kit to the manufacturer’s specifications, eluting with 100 μL nuclease-free water. The concentration of RNA was determined using either a Thermofisher Nanodrop 1000 or a Denovix DS-11+ spectrometer. All RNA was then stored at −80 °C until use.

### 2.6. Pathogen Quantification

Pathogen quantification was accomplished using one-step reverse-transcription qPCR (rt-qPCR). The sequences used are listed in Table 1. The primer for *B. burgdorferi* targets the 16 s rRNA gene. The primer for POWV targets the NS5 gene of POWV, which is one part of the single piece of RNA that makes up the POWV genome.

The reactions utilized an iTaq Universal SYBR one-step kit (Biorad) to both generate cDNA and perform qPCR in an uninterrupted process. Each reaction used 10 μL of master mix, 0.5 μL of the reverse-transcriptase enzyme, 0.3 μL of nuclease-free water, and 1.2 μL of primer solution. The primer solution contained 10 μM of both the forward and reverse primers. The sample volume per reaction was 8 μL. The reactions were run on either a Biorad IQ-5 or Biorad CFX96 machine. For each machine used, an individual standard curve was run to correlate Ct outputs from that particular machine against RNA derived from the viral or bacterial stock of known concentration. The cycle parameters consisted of 10 min at 50 °C followed by 3 min at 95 °C for the cDNA synthesis and iTaq preparation steps. The qPCR reading portion involved 45 cycles of 15 s at 95 °C and 30 s at 60 °C, which was the reading step. This was followed by an 81-cycle melt curve at 0.5 °C per cycle from 55 to 95 °C. Output Ct values were converted into log_10_(cells) or log_10_(FFU) using standard curves based on known pathogen quantities. Those values were then normalized against the mass of RNA added to each reaction as calculated by the spectrometric readings and the volume of sample added to each well.

The midguts were tested first. The midguts were considered positive if the Ct value was above the limit of detection of the qPCR assay as determined by the standard curve for each individual qPCR machine. Any value below the limit of detection was set to zero, representing no pathogen. Coinfected midguts were considered positive only if both POWV and *B. burgdorferi* were detected. All tested salivary glands belonged to ticks with positive midguts.

### 2.7. Statistics

Statistical analysis was performed using R 3.5.2. For the midgut, sex and infection categories were considered against pathogen quantity in a set of two two-way ANOVAs, one for POWV quantity in log_10_(FFU/μgRNA) and the other for *B. burgdorferi* quantity in log_10_(cells/μgRNA). Ticks that were not successfully infected, as determined by the qPCR limit of detection for the relevant pathogens, were excluded from the analysis. Significance within individual categories was identified using Tukey’s *post hoc* test. In the salivary glands, successfully infected ticks were grouped and compared between the POWV-only and coinfected groups by a Student’s *t*-test. *p*-values of less than 0.05 were considered significant, and *p*-values of less than 0.01 were considered especially significant.

### 2.8. Illumina Next-Generation Sequencing

Twenty-four ticks were selected for next-generation RNA sequencing analysis. These included eight ticks from each test group (control, *Borrelia*-only, POWV-only, and coinfected). Each group contained four male and four female ticks, selected to have similar pathogen loads in the midguts and salivary glands. The midgut and salivary glands of every selected tick were used, making a total of 48 samples selected for sequencing. The RNA samples were analyzed using an Agilent 2100 Bioanalyzer. Ticks with a sample having inadequate RNA quantity or quality were replaced were exchanged with equivalent ticks of the same sex and test group with similar pathogen load in the midgut and salivary glands.

The RNA was sequenced using an Illumina Next Generation Sequencing approach. RNA-seq libraries were constructed using Illumina TruSeq V2 kits and then processed with an Illumina Hiseq 2000 machine. The sequencing process generated about 10 million reads per sample, which was below the manufacturer-recommended target of 15–20 million reads. The samples, therefore, underwent a second round of sequencing, generating about 14 million reads. These were combined with the results of the first run. Sequence analysis was performed using Partek Flow (Partek). The reads were trimmed to remove the specific sequences added during library preparation then aligned using the Bowtie 2 algorithm against the genome of *I. scapularis* (*Ixodes scapularis* Wikel genome, IscaW1.6). Sequences that successfully aligned with the genome were processed through an annotated transcriptome model to identify the specific genes by their ISCW number. The data were normalized by quantile and the difference between pathogen-infected categories versus the uninfected control calculated as a fold-change. The statistical significance of each fold-change from control was determined based on a two-way ANOVA considering sex and test group.

Transcripts with fold changes of less than 1 but greater than −1 were excluded from analysis, as well as those with *p*-values greater than 0.05. A list of the products of the transcripts and the origin of their identification was constructed by searching the tick transcriptome housed at Vectorbase.org using a program constructed in R 3.5.2.

For each infection category and organ (Borrelia-only, POWV-only, and co-infection for midguts and salivary glands), the ISCW numbers for significantly up- and down-regulated genes were imported into g:Profilier [43] to assess the functional consequences of change in transcription. For this, up- and down-regulated transcripts for each infection category were submitted independently to create lists of upregulated and downregulated functions.

## 3. Results

### 3.1. Capillary Inoculation Successfully Introduced POWV and B. burgdorferi into the Tick Midgut

In the *Borrelia*-only group, 52 ticks were attempted, and 23 midguts were successfully infected (40%; 16/36 female and 7/22 male). In total, 59 ticks were exposed to POWV-only, and 38 developed midgut infection (61%; 22/34 female, 14/25 male). Of 79 ticks in the co-infection group, 18 were successfully infected with both pathogens (23%; 13/52 female and 5/27 male). These results are summarized in Table 2. The observed co-infection rate was almost identical to the predicted rate of co-infection based on the product of the *Borrelia*-only and POWV-only groups (24% all).

### 3.2. The Presence of B. burgdorferi in the Midgut Increases POWV Replication

The quantity of POWV and *B. burgdorferi* in successfully infected ticks was compared by two-way ANOVA to determine statistically significant increases or decreases in pathogen quantity in response to co-infection. The two-way ANOVA considered both infection categories (*Borrelia*-only, POWV-only, and co-infection) and tick sex (male and female).

**Figure 1:**
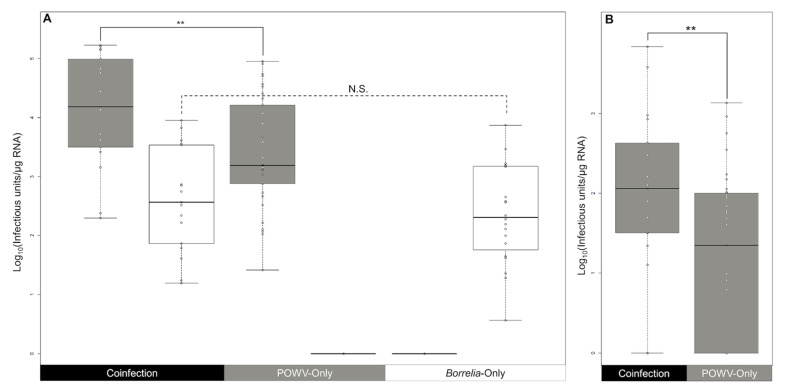
(**A**) POWV (gray) and *B. burgdorferi* (white) quantities detected in the midguts of capillary-inoculated ticks as determined by qPCR quantification. Statistically significant comparisons are marked with “**” for *p*-values less than 0.01, and N.S. for *p*-values greater than 0.05. A statistically significant increase in POWV (*p* = 0.009) was observed between the POWV-only group (*n* = 33) and simultaneously coinfected groups (*n* = 17). No statistically significant chance in *B. burgdorferi* (*p* = 0.464) was observed between the coinfected and *Borrelia*-only group (*n* = 20). (**B**) POWV observed in the salivary glands of POWV-only (*n* = 33) and coinfected (*n* = 17) group ticks with infected midguts. The quantity of POWV in the salivary glands of all ticks with infected midguts shows a statistically significant (*p* = 0.008) increase in POWV quantity in coinfected salivary glands.

A statistically significant increase was found in the quantity of POWV in the midgut in response to co-infection with *B. burgdorferi* (*p* = 0.009). This difference was statistically significant in all ticks and also statistically significant in male ticks. The difference in the quantity of *B. burgdorferi* in the midguts of *Borrelia*-only ticks versus coinfected ticks was not statistically significant (*p* = 0.464). These results are displayed in Figure 1A. These results indicate that the presence of POWV does not increase or decrease the amount of *B. burgdorferi* present in the tick midgut but that the presence of *B. burgdorferi* in the midgut increases the replication of POWV within the first three weeks of infection.

### 3.3. Capillary Inoculation Produces Dissemination of POWV to the Tick Salivary Glands

The salivary glands of ticks with successfully infected midguts were screened for POWV and *B. burgdorferi*. In the POWV-only group, 33 salivary glands were tested. Of these, 20 ticks (61%) showed POWV dissemination to the salivary glands (12/19 female, 8/14 male). In the coinfected group, 14 of the 17 salivary glands tested were infected with POWV (12/12 female, 2/5 male). These percentages are organized in Table 3.

### 3.4. The Presence of B. burgdorferi in the Midgut Increases the Quantity and Dissemination of POWV to the Salivary Glands

In the POWV-only group, the dissemination rate in both male and female ticks was 61% (20/33), while in coinfected ticks the observed rate was 88% (15/17) with 100% (13/13) detection of POWV in female ticks with *B. burgdorferi* present in the midguts (Figure 1A). Since only four of these ticks had detectable *B. burgdorferi* in the salivary glands, the increased rate of POWV dissemination appears to be independent of spirochete dissemination and instead occurs in response to the presence of *B. burgdorferi* in the midgut. In comparing the quantity of POWV in the salivary glands of all ticks in the POWV-only and coinfected groups with successfully infected midguts, a statistically significant increase in the quantity of POWV was identified in the coinfected group (*p* = 0.01), which is shown in Figure 1B. Therefore, not only does the presence of *B. burgdorferi* in the midgut increase the replication of POWV in the midgut, but it also increases the rate of dissemination of POWV to the salivary glands independent of the dissemination of *B. burgdorferi*. This suggests that the reaction of the midgut to the presence of *B. burgdorferi* creates a response within the tick that promotes replication of POWV throughout the tick.

### 3.5. Midgut Transcriptome Shows Borrelia-Directed Response to Infection

**Figure 2:**
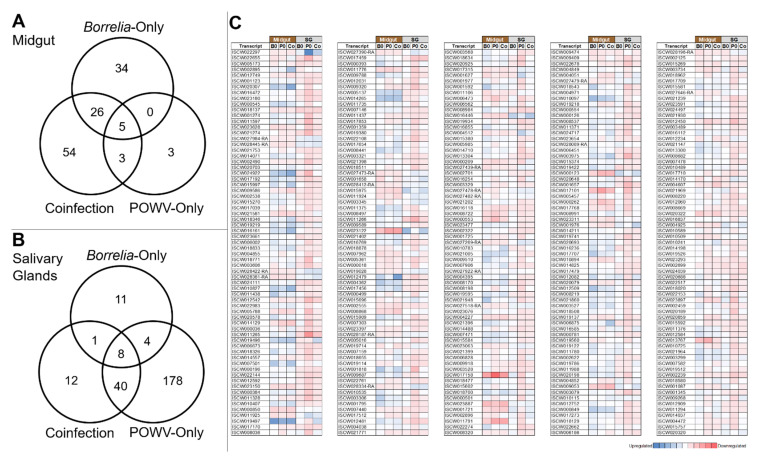
Diagrams showing the number of statistically up- or down-regulated genes from control in ticks infected with only *B. burgdorferi*, only POWV, or ticks infected with both, as well as genes shared between multiple categories. These include genes in the midgut (**A**), where the presence of POWV produces a minimal response compared to the *Borrelia*-only and coinfected groups, and the salivary glands of ticks with infected midguts (**B**), which show a strong response to POWV but a weaker response to co-infection or *Borrelia*-only infection. (**C**) Heatmap of midgut and salivary gland transcripts in response to infection with *B. burgdorferi* only (B0), POWV only (P0), or a combination of the two pathogens (Co). All transcripts in the represented in the heatmap were significantly altered (*p* < 0.05) from control expression in the co-infection category. All transcripts were significantly altered (*p* < 0.05) from control expression in the co-infection category.

The transcripts associated with each test group are displayed in the Venn diagram in Figure 2A and the heatmap in Figure 2C. Overall, comparatively few transcripts differed substantially from the control. The effect observed in response to POWV infection was remarkably low, with only 11 transcripts significantly changed in response to infection and only three of these unique to POWV alone.

Pathway analysis was performed to identify specific pathways and functions associated with the specific up- and down-regulated genes of the midgut in each infection category. In the case of the POWV-only group, the only function to have statistically significant downregulation was G-protein coupled glutamate receptor binding (*p* = 0.05). No pathways were significantly upregulated. This relates directly to the low differential response observed in POWV-infected midguts, with only 11 transcripts demonstrating significant differences from control expression and only three of these being unique to the POWV-infection group alone (Figure 2A). These include eight downregulated transcripts and three that are upregulated (Supplementary Figure S1B).

The response was markedly different in cases where *B. burgdorferi* was present in the midgut. In the *Borrelia*-only group, 65 transcripts were identified as being significantly altered versus the control. Of these, 21 were downregulated and 44 were upregulated. Based on an analysis of the pathways represented, this upregulation was closely tied to protein binding, folding, and synthesis (Supplementary Figure S3A), with the downregulated transcripts suggesting that there are no statistically significant pathways represented. Based on the assigned functions of the genes (Supplementary Figure S1A), it is apparent that many of these also have roles in general metabolism, transcription, and biomolecule synthesis.

The transcriptional response of the midgut to co-infection is similar to the response to infection with *B. burgdorferi* alone. In the coinfected group, 88 transcripts differed significantly from control midguts, with 71 upregulated and 17 downregulated (Supplementary Figure S1C). Of these, 31 (35%) were shared with the *B. burgdorferi* group and only 8 (9%) shared with the POWV-only group. This demonstrates similarity to the *Borrelia*-only response, although numerous transcripts unique to this category were also altered. Analysis of the altered pathways indicates an increase in electron transfer within the electron transport change, vesicle formation and use, and protein folding (Supplementary Figure S3B). The only downregulated function involved the G-protein coupled glutamate receptor signaling pathway, a feature shared with the POWV-only group. The functional changes are, therefore, most similar to those observed in *Borrelia*-only infection, with overlap with the POWV-only group being reduced due to the small number of transcripts altered by POWV-only infection.

### 3.6. Salivary Gland Transcriptome Shows POWV-Directed and Borrelia-Attenuated Response to Infection

The RNA samples from the salivary glands underwent an identical sequencing process to those derived from the midgut. Significantly up- and down-regulated transcripts in response to infection categories were identified. The results of this analysis are displayed in the Venn diagram and heatmap in Figure 2B and the heatmap in Figure 2C.

Unlike in the midgut, the salivary gland response to infection with POWV was substantial. A total of 230 significantly, with 178 of these being unique to POWV single infection. The majority of these genes (210) were down-regulated, with 20 being up-regulated. The down-regulated transcripts have numerous functions related to aerobic respiration, ATP synthesis, the electron transport chain, proton channel activity, and ribosomal synthesis (Supplementary Figure S3C). The only significantly upregulated pathways are associated with a small number of transcripts relating to the regulation of phosphatase. In the salivary glands of POWV-infected ticks, therefore, there is a significant decrease in energy production as well as ribosomal construction and a down-regulation of basic metabolic processes.

This major shift is not shared with the *Borrelia*-only group, which experienced a statistically significant change in only 24 transcripts (8 down-regulated, 16 up-regulated). Of these, 24, 12 (50%) are shared with the POWV-only group (Figure 2B). Functionally, only the G-protein coupled glutamate receptor binding pathway was up-regulated in this group (*p* = 0.05). The response to *B. burgdorferi* alone is minimal. In contrast to the transcriptional profile of the midgut, the transcriptional changes in the salivary glands are most similar between the POWV-only and co-infection categories. In the salivary glands of ticks with *B. burgdorferi* and POWV present in their midguts, the expression of 61 transcripts (21 up-regulated, 40 down-regulated) was significantly altered versus control ticks (Supplementary Figure S1B), with 48 of these transcripts shared with the POWV-only group (Figure 2B). Functionally, the coinfected salivary glands have evidence of decreased ribosomal synthesis and slightly increased NADPH-related enzymes (Supplementary Figure S3D). Therefore, in addition to consisting of many of the transcripts shared with the POWV-only group, some of the down-regulated functional pathways present are also maintained.

The response to co-infection of the salivary glands was most similar to that of POWV, with 48 genes shared between the coinfected group and the POWV-only group. The POWV-only and coinfected salivary gland sequencing samples used in this portion of the study were both infected with POWV, but despite this, the response to co-infection involves only 27% of the transcripts that are significantly changed in the POWV-only category. The response, therefore, is substantially reduced when *B. burgdorferi* is present in the tick midgut. Functionally, the pathways decreased in the POWV-only group but not in the co-infection group are related to respiration, metabolism, and energy generation, suggesting that the salivary glands of coinfected ticks do not experience the same broad metabolic shutdown as ticks with POWV alone.

## 4. Discussion

Male and female *Ixodes scapularis* ticks were orally inoculated with POWV, *B. burgdorferi*, or a combination of the two and allowed to incubate for three weeks. The ticks were then dissected, and RNA was extracted from the midguts and salivary glands of these ticks. The POWV and *B. burgdorferi* quantities within the organs of the ticks were then determined by qPCR. The RNA additionally underwent next-generation RNA-sequencing to identify significantly altered transcripts between infected ticks and ticks fed uninfected media by the same capillary-feeding process.

### 4.1. Midgut Infection and Response

In the coinfected tick midgut, the presence of *B. burgdorferi* resulted in a statistically significant increase in the normalized quantity of POWV versus ticks infected with only POWV. RNA-seq analysis was performed to identify the source of this interaction. In the midgut, the tick transcriptional response to POWV-only infection is remarkably minimal, with only 11 transcripts significantly altered from control and five of those shared between the *B. burgdorferi* and co-infection groups, suggesting that those are generalized responses to infection with either pathogen.

In the midgut, the primary response to infection in POWV/*B. burgdorferi* infected ticks is driven not by POWV but rather by *B. burgdorferi*. Normally, *B. burgdorferi* spirochetes reside affixed to the epithelial lining of the tick midgut on the luminal side. This site is immunologically privileged, and the majority of spirochetes remaining in that area are safe from attack by the tick’s immune system. However, some spirochetes do prematurely escape the midgut and enter the tick hemocoel [44], where they are quickly destroyed by tick hemocytes and granulocytes [32]. The escape of spirochetes may then allow for immune activation, either of resident immune cells within the midgut or due to the infiltration of tick immune cells into the tissue. This would then be responsible for the observed increase in protein-handling functions, as well as the overall metabolic increase observed within the midgut during *B. burgdorferi* infection.

The response of the midgut to POWV-only infection was remarkably minimal, despite POWV replication having been detected in the midgut tissue of these ticks. This may be due to an inherent inability of the midgut to detect and respond to viral-associated molecular patterns or due to the suppression of the system by unidentified molecular features of the virus itself. It is notable that POWV infection does not cause an apparent loss of tick fitness or a decrease in tick survival, which is evolutionarily consistent with a virus that utilizes a long-lived arthropod vector.

The midgut response to co-infection is similar to the response to *Borrelia*-only infection, suggesting that the midgut response is driven primarily by the presence of *B. burgdorferi* and not by POWV. Although the response is functionally similar, with increases in protein-handling, vesical production, and metabolic aspects, it is also driven by a dissimilar set of transcripts, further indicating that while POWV infection alone does not induce a substantial change within the midgut, its presence does alter the midgut’s response to the presence of *B. burgdorferi*.

This response in the *Borrelia*-only and co-infection groups causes an increase in midgut cellular activity, likely due to immune infiltration. This process is not harmful to *B. burgdorferi* in the unfed tick as, at this stage, the spirochetes destined for transmission are still residing on the luminal side of the midgut where they are protected, hence why there was no statistically significant variation in the amount of *B. burgdorferi* present in the tick midgut in response to co-infection with POWV. POWV, though, actively replicates throughout the midgut. It has also been observed to utilize mammalian macrophages as an early host cell during infection [40], and this may relate to a parallel process within the tick phagocytes. The more active midgut was observed during *B. burgdorferi* infection, especially with the possible infiltration of immune cells, which provides POWV with better conditions for more rapid replication and dissemination throughout the tick. POWV, in turn, enhances and potentiates this response.

Other studies have indicated that *B. burgdorferi* in *I. scapularis* or *B. afzelii* in *I. ricinus* produce transcriptional effects within the midgut of ticks. These include sizable metabolic changes associated with mitochondrial proteins and chitinase, both of which are associated with the formation of the peritrophic membrane [38]. This process is more involved with feeding, though, a process that is ignored due to the use of capillary feeding for inoculation in this system. Several immune pathways are also involved, including the JAK-STAT and immune-deficiency (IMD) pathways [36], and additional anti-microbial genes activated through PIXR and its associated pathways [37]. Some immune transcripts were identified in the midgut in this study, namely, a lipopolysaccharide-induced transcription factor for regulating tumor necrosis factor-alpha (ISCW020307), upregulated in both the *Borrelia*-only and co-infected groups, and a 65-kDa macrophage protein (ISCW012584) in the *Borrelia*-only group.

### 4.2. Salivary Gland Infection and Response

The salivary glands of ticks with infected midguts were tested for the presence of POWV to determine changes in viral dissemination in response to co-infection.

The rate of POWV dissemination to the salivary glands was enhanced by the presence of *B. burgdorferi* in the midgut. This was especially true of female ticks, where 100% of the ticks with coinfected midguts had detectable POWV in the salivary glands instead of 63% in the POWV-only group. Comparing the quantity of POWV in salivary glands of POWV-only and coinfected ticks with successfully infected midguts, a statistically significant increase in POWV quantity was observed. This correlates to the response observed in the midgut. However, the coinfected salivary glands did not universally contain *B. burgdorferi*, suggesting the response is not induced by the local presence of the spirochetes but rather by a systemic response to those escaping the midgut.

RNAseq analysis of the salivary glands revealed a highly dissimilar situation in the midguts. While POWV produced only a small transcriptional response in the midgut, the response in the salivary glands was substantial. Further, the response to co-infection in the salivary glands is functionally partially similar to the response to POWV-infection alone. The magnitude, however, is reduced; the response in co-infection contains only 27% of the number of transcripts shared with the POWV-only group. The presence of *B. burgdorferi* in the tick midgut, therefore, reduces the transcriptional response to POWV infection, especially in functional categories related to cellular respiration and energy metabolism, with functions associated with decreased ribosomal synthesis and function persisting regardless of the persistence of *B. burgdorferi*. Since the inhibition of these processes results in greater dissemination and quantity of POWV in the salivary glands of coinfected ticks, this response appears to have an immune function and, consequently, the presence of *B. burgdorferi* in the midgut is immunosuppressive.

The metabolic reduction observed in POWV-only ticks reduces cellular metabolism and protein assembly, a process that may be a response to replicating POWV. The salivary gland is the target organ of infection for POWV within the tick and is required for its transmission. The reduction of the metabolic function corresponds to a reduction in POWV replication. It may be one of the tick’s primary means of limiting viral replication in an unfed state when the function of the salivary gland is not immediately critical to tick survival. POWV infection may also change the expression of salivary components to modulate the host response to infection as observed for TBEV [40], a process that would now be altered by the presence of *B. burgdorferi* in the midgut.

These observations differ somewhat from other studies examining the impact of infection of the salivary glands, although this may be because the focus of many salivary gland studies is more focused on secreted salivary factors and salivary components rather than on transcripts for metabolic processes. Infection of *I. ricinus* salivary glands with *B. afzelii* has been shown to increase the expression of salivary products such as lipocalins and ixodegrin [38]. Similar effects have been observed when the salivary glands are infected with tick-borne Encephalitis virus (TBEV), where changes to lipocalins, Kunitz-domain protease inhibitors, metalloproteases, and antimicrobial proteins were observed [39,40]. Additional responses in tick cell lines were observed, including changes to heat-shock proteins, histones, metabolic proteins, and transcripts associated with the tick’s innate immune response [41]. The RNAi knockdown of heat-shock proteins in the cell lines was found to increase the replication of the Langat virus, suggesting that they do indeed have a role in the antiviral response. Here, heat-shock proteins were observed to be downregulated in the POWV-only group in this study (ISCW024922, ISCW017709, and non-significant downregulation in ISCW017456) along with downregulation of several immune-related transcripts such as calcineurin B (ISCW017170), and N-CAM immunoglobulin domain-containing protein (ISCW022144). Transcripts associated with metabolic functions were also observed. This suggests the possibility that the effects seen in this study may not be strictly associated with the saliva-secreting mechanism of the salivary gland, but rather with the organ as a whole, including its structural and supporting tissues. 

### 4.3. Limitations of the Capillary-Inoculation System

It is important to note that capillary inoculation imposes certain limitations on the results of this study. This process was selected due to its utility in delivering pathogens directly to the midgut without exposing other potential entry routes such as the trachea and genital pore as would be the case with synchronous inoculation. The intent was to replicate questing adult ticks, with *B. burgdorferi* present on the luminal surface of the midgut and POWV present in the midgut epithelia and possibly throughout the tick. In nature, the ticks would be infected during either their larval or nymphal feeding on a suitable reservoir, often *Peromyscus leucopus*. Under these circumstances, the consumption of blood has substantial effects both structurally and transcriptionally on the ticks. Physically, the midgut undergoes substantial enlargement, along with the development of a peritrophic membrane within the midgut during digestion, which adds an additional barrier to pathogen infection [36], an effect not modeled in this experiment. The ticks then molt to the next life stage, undergoing substantial anatomical remodeling and development in the process [45]. Transcriptionally, the presence of a bloodmeal causes a number of changes that can substantially alter the tick’s metabolic processes, in some cases changing the amenability of the tick to pathogen colonization [38], even with only a short duration of feeding [40]. These processes may have a substantial impact on pathogen acquisition. The purpose of this model was to assess pathogen interaction in unfed, questing adult ticks by recreating this situation artificially.

Additionally, both male and female ticks were utilized in this study. In nature, the nymphs of either sex are structurally and behaviorally indistinguishable, with secondary sexual characteristics and behaviors developing only after the nymphal feeding. Since the ticks can only transmit pathogens acquired during the larval or nymphal feedings, this suggests that the rate of infection in male and female ticks is similar. For *I. scapularis*, though, male ticks do not feed on vertebrate hosts as extensively as the females do. Therefore, while their biological response to co-infection may be similar to that of females, they are rarely responsible for the direct transmission of the pathogen to a host. This does not necessarily preclude their role in transmission, however. In *I. scapularis*, males that mate while the female is feeding often actively feed on the female during mating. During this process, it may be possible for infected males to transmit pathogens to the hemolymph of the female and then, in turn, to the parasitized host. The ability of this behavior to transmit male-derived pathogens, especially with regard to bacterial and viral co-infection, has not been studied extensively, and it is unclear whether or not it is an effective means of pathogen transmission.

## 5. Conclusions

These results demonstrate that POWV and *B. burgdorferi* can coexist in *I. scapularis* ticks without negative interaction and with a reduction in the replication of either pathogen. The replication and dissemination of POWV within the tick are enhanced by the presence of *B. burgdorferi* in the tick midgut. Therefore, the rate of co-infection in nature can be predicted to be at a minimum, the product of the rates of *B. burgdorferi* and POWV in a tick population. Due to this, most ticks infected with POWV can be predicted to be infected with *B. burgdorferi* based on its rate in the area. Without exhibiting competition for vector infection, neither pathogen can exclude the other from its geographical range and can coexist continuously, dependent on secondary factors in their respective sylvatic cycles. Humans inhabiting or traveling through such an area are in turn at risk for exposure to both pathogens from a single tick bite, and in fact, most cases of POWV transmitted by *I. scapularis* can be anticipated to have a Lyme component that may be undiagnosed. The exact clinical consequence of simultaneous human infection with *B. burgdorferi* and POWV is poorly understood. Still, considering the likelihood of coinfected ticks, this is most likely the dominant nature of POWV infection.

## Figures and Tables

**Table 1:** Primers used for the detection of *B. burgdorferi* and POWV by one-step, reverse-transcription qPCR in this experiment.

Target	Accession Number	Forward Sequence	Reverse Sequence	Source	Product Length
*B. burgdorferi* 16S	CP017201.1	CAGATAAGACT GCCGGTGATAAG	CCGGACTGAGACCTGCTTTA	[29]	156
POWV NS5	MK733761.1	CCGAGCCAA AGTGAGGATGT	TCTTTTGCCG AGCTCCACTT	Designed	156

**Table 2:** Midgut infection rates by test group and tick sex. Oral exposure to POWV resulted in successful midgut infection with a relatively high success rate, while *B. burgdorferi* infection by this method produced lower rates of infection. The co-infection rate is similar to the individual rates of infection for *B. burgdorferi* and POWV acquisition. The capillary inoculation method is an effective means of inoculating ticks with detectable quantities of *B. burgdorferi* and POWV in the midgut.

Group	All	Female	Male
*Borrelia*-only	(23/52) 40%	(16/36) 44%	(7/22) 31%
POWV-only	(38/59) 61%	(22/34) 65%	(14/25) 56%
Coinfected	(18/79) 23%	(13/52) 25%	(5/27) 19%

**Table 3:** Infection rates of salivary glands in ticks with successfully infected midguts, representing pathogen dissemination. *Borrelia burgdorferi* was observed in some salivary glands, although POWV dissemination was more common.

Group	*B. burgdorferi*-Infected			POWV-Infected		
	All	Female	Male	All	Female	Male
*Borrelia*-only	(4/20) 20%	(4/14) 29%	(0/6) 0%	(0/20) 0%	(0/14) 0%	(0/6) 0%
POWV-only	(0/33) 0%	(0/19) 0%	(0/14) 0%	(20/33) 61%	(20/33) 63%	(8/14) 57%
Coinfected	(4/17) 24%	(4/13) 31%	(0/4) 0%	(15/17) 88%	(13/13) 100%	(2/4) 50%

## Data Availability

Data obtained and discussed in this study are presented in this manuscript [main text and supplemental files].

## Supplementary Materials

The following supporting information can be downloaded at: https://www.mdpi.com/article/10.3390/v14071584/s1. Supplementary Figure S1: (**A**) Statistically significant transcripts of the *I. scapularis* midgut altered in response to infection with *B. burgdorferi*; (**B**) Statistically significant transcripts of the *I. scapularis* midgut altered in response to infection with POWV; (**C**) Statistically significant transcripts of the *I. scapularis* midgut altered in response to co-infection with *B. burgdorferi* and POWV. Supplementary Figure S2: (**A**) Statistically significant transcripts of the *I. scapularis* salivary glands altered in response to midgut infection with *B. burgdorferi*; (**B**) Statistically significant transcripts of the *I. scapularis* salivary glands altered in response to midgut and salivary glands with POWV; (**C**) Statistically significant transcripts of the *I. scapularis* salivary glands altered in response midgut co-infection with *B. burgdorferi* and POWV in addition to salivary gland co-infection with POWV. Supplementary Figure S3: Significantly altered functional pathways derived from the transcriptome of midguts of ticks infected with: (**A**) *B. burgdorferi* with the response primarily involved protein synthesis, binding, and folding pathways; (**B**) both POWV and *B. burgdorferi* with the response being functionally similar to the response to *B. burgdorferi* alone, with enhanced protein folding and operation, although with additional respiration and metabolic components; (**C**) significantly altered functional pathways derived from the transcriptome of the salivary glands of ticks with midguts and salivary glands infected with POWV, indicating extensive downregulation of various metabolic and respiratory functions, as well as downregulation of ribosomal biosynthesis pathways; and (**D**) altered pathways from the salivary glands of ticks with POWV-infected salivary glands and *B. burgdorferi* and POWV-infected midguts; this response is most similar to that of salivary glands infected with only POWV, with downregulated ribosomal synthesis, but lacking significant downregulation of respiratory and energy-metabolism pathways.
