# Supplementary figures and images for: Infection with Borrelia burgdorferi Increases the Replication and Dissemination of Coinfecting Powassan Virus in Ixodes scapularis Ticks
Source: Viruses. 2022 Jul 21;14(7):1584. doi: 10.3390/v14071584 (PMC9319581; doi:10.3390/v14071584)

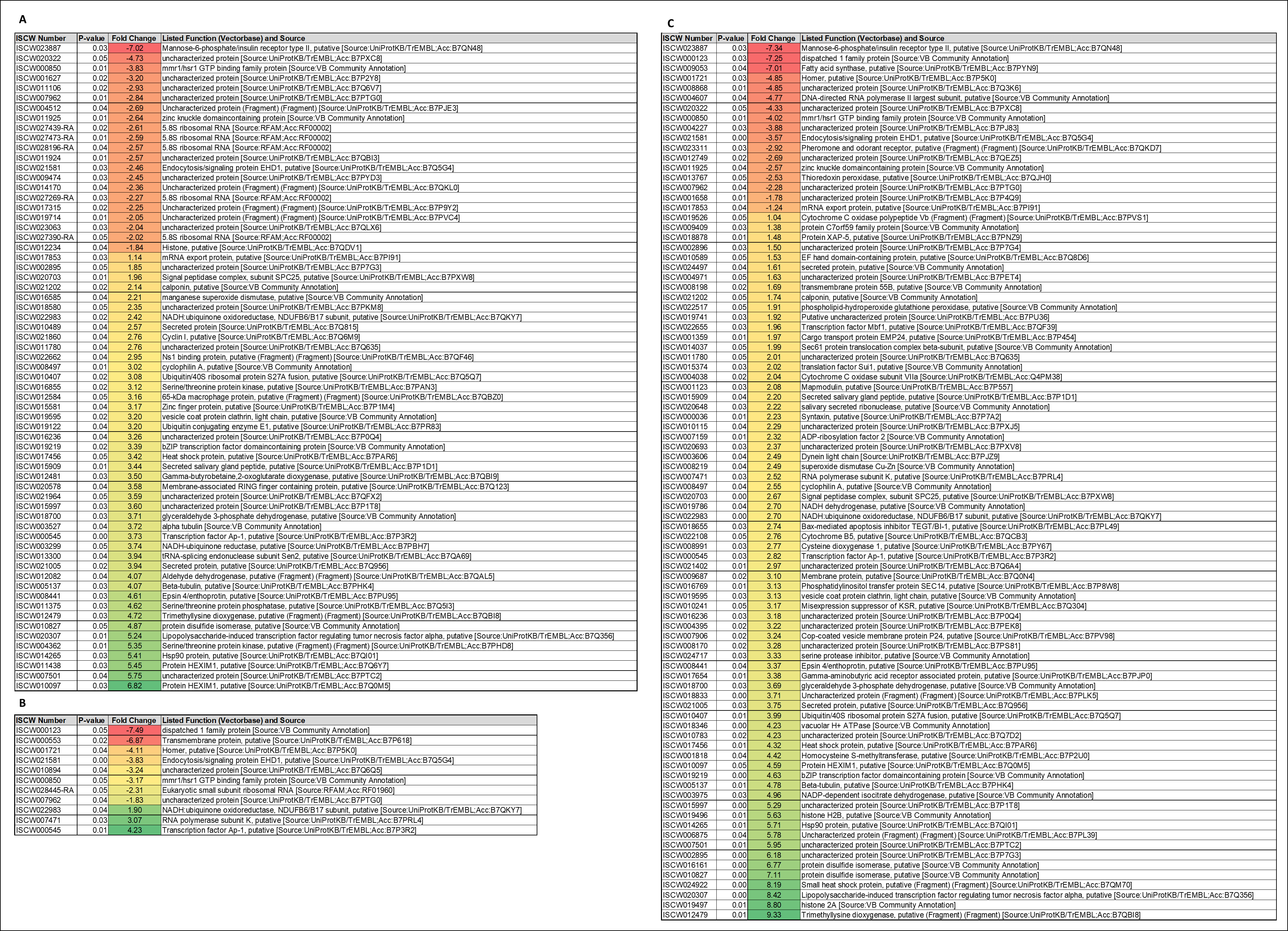

Supplement: Supplementary file 1 [file viruses-14-01584-s001.zip › SupFIGURE1.tiff]

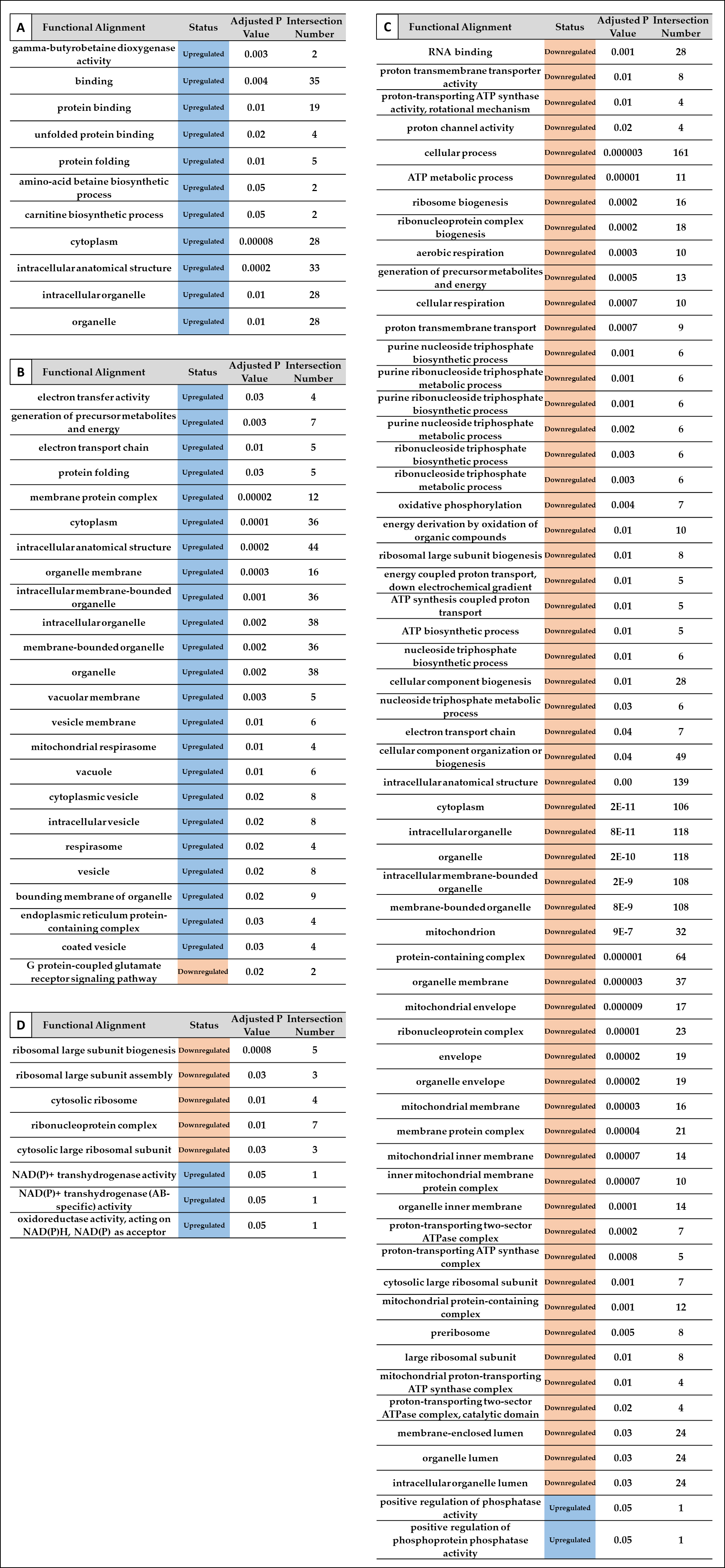

Supplement: Supplementary file 1 [file viruses-14-01584-s001.zip › SupFIGURE3.tiff]
